# Theta band activity in response to emotional expressions and its relationship with gamma band activity as revealed by MEG and advanced beamformer source imaging

**DOI:** 10.3389/fnhum.2013.00940

**Published:** 2014-02-03

**Authors:** Qian Luo, Xi Cheng, Tom Holroyd, Duo Xu, Frederick Carver, R. James Blair

**Affiliations:** ^1^Department of Neurosurgery, Saint Louis University School of MedicineSaint Louis, MO, USA; ^2^Unit of Affective Cognitive Neuroscience, National Institute of Mental HealthBethesda, MD, USA; ^3^The Lieber Institute for Brain Development, Johns Hopkins Medical CampusBaltimore, MD, USA; ^4^MEG Core Facility, National Institute of Mental HealthBethesda, MD, USA

**Keywords:** MEG, theta, gamma, emotion, event-related synchronization

## Abstract

Neuronal oscillations in the theta and gamma bands have been shown to be important for cognition. Here we examined the temporal and spatial relationship between the two frequency bands in emotional processing using magnetoencephalography and an advanced dynamic beamformer source imaging method called synthetic aperture magnetometry. We found that areas including the amygdala, visual and frontal cortex showed significant event-related synchronization in both bands, suggesting a functional association of neuronal oscillations in the same areas in the two bands. However, while the temporal profile in both bands was similar in the amygdala, the peak in gamma band power was much earlier within both visual and frontal areas. Our results do not support a traditional view that the localizations of lower and higher frequencies are spatially distinct. Instead, they suggest that in emotional processing, neuronal oscillations in the gamma and theta bands may reflect, at least in visual and frontal cortex either different but related functional processes or, perhaps more probably, different computational components of the same functional process.

## INTRODUCTION

Neuronal activities of the brain are characterized by rhythmic oscillations with a wide range of frequencies (from less than 0.05 Hz to more than 500 Hz; Buzsaki and Draguhn, 2004). Neuronal oscillations are suggested to provide temporal and spatial codes, and to act within communication networks to coordinate distinct neural processes into highly ordered cognitive functions (Singer, 1999; Varela et al., 2001; Fries, 2005). However, despite a considerable amount of research on the functions and properties of neuronal oscillations in various frequency bands, our knowledge regarding the functional relationship between oscillations across different frequency bands remains limited. Recent data suggest that cross frequency interaction might be a neuronal process widely seen in many different processes and across different areas of the cortex and (Jensen and Colgin, 2007; Lisman and Buzsáki, 2008 for a review; Bruns and Eckhorn, 2004; Canolty et al., 2006; Lakatos et al., 2008).

The relationship between theta and gamma band activity during cognitive and emotional processing has been debated. A traditional view holds that the localization of lower and higher frequencies should be spatially distinct. It is argued that the brain is comprised of several phylogenetically distinct networks and each network has its own natural frequency by which the network operates. Slow wave activity is thought to stem from the “evolutionary older” subcortical structures while fast wave activity is thought to originate from the more complex cortical structures (MacLean, 1990; Michel et al., 1992; Robinson, 2000, 2001; Knyazev and Slobodskaya, 2003; Knyazev et al., 2004). However, some recent studies reveal that gamma and theta are not spatially distinct but can be both present in the brain regions implicated in a task but with different temporal dynamics (e.g., Belluscio et al., 2012). Thus, a recent study indicates the presence of both theta and gamma activity in the hippocampus during maze learning; moreover, the oscillators at the gamma and theta frequencies were interdependent, leading to the suggestion that cross phase coupling can integrate multiple layers of information to support multiple time-scale control of neuronal spikes within and across structures (Belluscio et al., 2012).

Little has been done to compare gamma and theta oscillations in human emotional processing. However, emotional processing is an interesting test case as it frequently implicates both cortical and subcortical structures (Blair et al., 1999; Luo et al., 2007, 2010; Pessoa and Adolphs, 2010; Levens et al., 2011). In previous work, we reported gamma band activity within the amygdala, visual cortex and inferior frontal gyrus (IFG)/insula in response to emotional stimuli (Luo et al., 2007). The goal of the current paper was to report the results of a re-analysis of these data focusing on theta band activity. We predicted that if there are region specific natural frequencies (MacLean, 1990; Michel et al., 1992; Robinson, 2000, 2001; Knyazev and Slobodskaya, 2003; Knyazev et al., 2004), then subcortical structures (e.g., the amygdala) would be more associated with theta band activity while cortical structures would be more associated with gamma band activity, e.g., IFG, anterior cingulate cortex (ACC), and orbital frontal cortex (OFC). Even if this was not the case, we were interested in determining whether activity in the theta vs. gamma bands within these regions was temporally synchronous or distinct.

Here we employed magnetoencephalography (MEG), an advanced beamformer source analysis technique synthetic aperture magnetometry (SAM) based on the beamformer approach (Vrba and Robinson, 2001; Hillebrand et al., 2005) in combination with the sliding window analysis (see Luo et al., 2007, 2009, 2010). These methods not only provide frequency-specific oscillatory power changes but also enable us to determine the dynamic spatiotemporal profiles of event-related oscillations.

## EXPERIMENTAL PROCEDURES

### DESIGN AND DATA ACQUISITION

The same data collected from our previous study (Luo et al., 2007) were used for the present study. In Luo et al. (2007), only the gamma band data were analyzed. In the present study, we analyzed the theta band data and compared results between the gamma and the theta band.

Briefly, fifteen subjects participated in the study. The stimuli were faces with fearful, angry and neutral expressions. Each face was presented for 300 ms followed by a 200 ms blank screen. There were 52 trials for each of the three conditions. The subject made a gender judgment after seeing the subsequent 1500 ms response window, which contained either “M F” or “F M”. The response was made based on the relative position of the letters “F” and “M”. The position of “M” and “F” was randomized across trials. For example, if the face was a female one and the subsequent window was “F M”, then they should press the left button; whereas if the response window was “M F”, the right button. This separation of the stimulus from the response window was made to reduce the subject’s expectancy and preparatory responses. The response window was followed by a blank screen for 600 ms.

The MEG data were recorded at 600 Hz using a 275-channel CTF whole head MEG system in a shielded environment with 3rd gradient balancing to reduce external noise. High-resolution anatomical images were acquired using a T1-weighted, three-dimensional, Spoiled GRASS imaging (spgr) sequence.

### DATA PROCESSING

The CTF software and software developed at the NIMH MEG Core Facility in combination with analysis of functional neuroimages (AFNI)^1^ were used for MEG/Magnetic resonance imaging (MRI) data processing. The DC (direct current) offset was removed, and the data were high-pass filtered at 0.61 Hz and powerline-notch filtered at 60 Hz (width = 3.1 Hz). The data were then marked according to the three stimulus types. A multisphere head model was created for each subject based on anatomical images of each subject using AFNI.

Data processing of gamma band was done in Luo et al. (2007). Briefly, to analyze task-related activation differences in the gamma frequency band (30–50 Hz), a sliding window SAM analysis was performed. With a window length of 150 ms and a step of 10 ms, we estimated the signal power in each voxel by using dual-state SAM imaging, in which the control state (baseline) was the 150 ms before stimulus onset (or -150–0 ms) and the active state was a 150 ms window sliding with a 10 ms step. Fifty dual-state SAM imaging analyses were performed with a spatial resolution of 7 mm. The output results were then concatenated, enabling us to obtain time courses in combination with spatial activation maps across all the time points starting from 150 ms before the stimulus to 500 ms after the stimulus. For group analysis, individual anatomical images were first spatially normalized to the Talairach brain atlas. The SAM results for different subjects were also normalized (transformed to z-scores) and then registered to their anatomical Talairach images. The group analysis for each frequency band and for each time window was then performed using a random-effects ANOVA model in AFNI, which generates the event-related oscillation results for the three conditions and the contrast effect between the three conditions. Fifty ANOVAs were performed. Voxels with an uncorrected *p* < 0.05 were considered statistically significant. A relatively lower *p* was adopted in order to observe the continuous spatiotemporal development. It should be noted though that for some regions, the highest peak power was lower than 0.0001.

Data processing in the theta band (5–8 Hz) was similar to that in the gamma band [e.g., all data processing was performed in a whole brain voxel-wise approach instead of an regions of interest (ROI) approach]. However, when doing SAM analysis in the theta band, a longer window length of 300 ms was adopted (in the gamma band the window length was 150 ms) with a 10 ms step. This was because low frequencies require longer observation times. However, different window lengths would **not** contribute to the difference in the event-related oscillation results between the two bands in the present study since the power values obtained from each window were averages over all the time within the window. Moreover, the power values were normalized for the window length. We estimated the signal power in each voxel by using dual-state SAM imaging, in which the control state (baseline) was the 300 ms before stimulus onset (or -300–0 ms) and the active state was a 300 ms window sliding with a 10 ms step: -300–0 ms, -290–10 ms, -280–20 ms, …, 200–500 ms. With sliding window SAM, we were able to tell quite accurately at what time significant event-related synchronization (ERS) emerges, peaks, and offsets. For example, if an ERS in a region did not reach the peak in the window beginning at –100 ms and ending at 200 ms, but peaked in the window beginning at –90 ms and ending at 210 ms, then we could conclude that the ERS in this region peaked between 200 and 210 ms. The output of SAM imaging analysis results were then concatenated, enabling us to obtain a time course in combination with spatial activation maps across all the time points starting from 300 ms before the stimulus to 500 ms after the stimulus. The high-performance computational capabilities of the NIH Biowulf Linux cluster^2^, was utilized to perform the above computation-intensive tasks. Group analysis was similar with that in the gamma band. Voxels with an uncorrected *p* < 0.05 were considered statistically significant.

Finally, we did a comparison of data from the gamma and theta bands. ERS peak latencies were contrasted for areas showing significant ERS in both bands. First, the ERS peak latency in these areas in the two bands was extracted from each individual subject. Then we did a *t*-test on the ERS peak latencies in the two bands for each of the regions.

## RESULTS

Our MEG-SAM results revealed significant ERS in the brain in the theta and gamma frequency bands in all the three conditions. See **Table 1** for details of spatiotemporal information for brain regions showing significant ERS in both the theta and the gamma bands.

**Table 1:** Spatiotemporal information of significant ERS in the gamma and theta bands.

Structure	Expression	L/R	BA	Band	Peak time	*x* (at the peak time)	*y* (at the peak time)	*z* (at the peak time)	*t* (at the peak time)
Amygdala	Fear	R		Gamma	230–240 ms	18	-8	-8	3.40
Amygdala	Fear	R		Theta	230–240 ms	13	-12	-13	3.2
Visual cortex	Fear	R/L	17/18/19/37	Gamma	140–150 ms	7	-88	-8	6.53
Visual cortex	Fear	R/L	17/18/19/37	Theta	200–210 ms	-18	-98	-8	4.12
Visual cortex	Anger	R/L	17/18/19/37	Gamma	140–150 ms	-6	-92	2	8.65
Visual cortex	Anger	R/L	17/18/19/37	Theta	260–270 ms	-18	-98	13	4.03
Visual cortex	Neutral	R/L	17/18/19/37	Gamma	140–150 ms	-2	-81	2	9.42
Visual cortex	Neutral	R/L	17/18/19/37	Theta	250–260 ms	3	-93	13	3.8
Insular	Fear	R	47/13	Gamma	240–250 ms	33	35	-8	3.37
IFG-Insular	Fear	R	44/47/13	Theta	330–340 ms	48	3	13	2.42
OFC	Anger	L	10	Gamma	200–210 ms	-18	43	-2.5	2.73
OFC–ACC	Anger	L	10/32	Theta	440–450 ms	-17	48	17	4.44

### THE AMYGDALA

**FIGURE 1:**
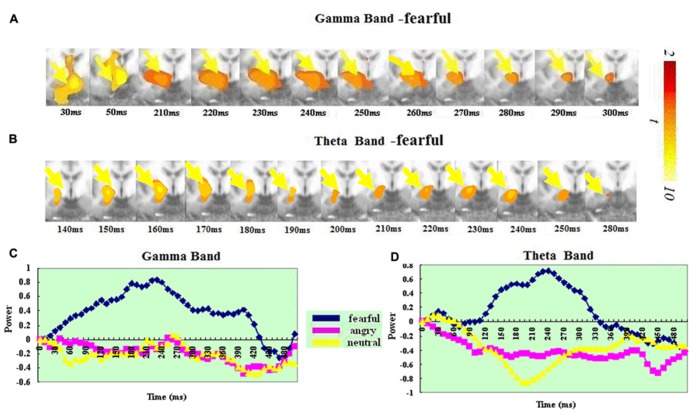
**Spatiotemporal profiles for the amygdala in the gamma and theta bands.** The brain map series **(A,B)** indicate the ERS development in specific brain areas with the left hemisphere is on the right and the right hemisphere is on the left. The curves **(C,D)** represent temporal profiles obtained from a peak voxel in a specified area in the three conditions respectively. The time courses are obtained from the normalized power value in each window. The blue curve refers to responses to fearful, the purple to angry and the yellow to neutral expressions. The X-axis represents the development of time (ms) with an increment of 10 ms for each time segment. The Y-axis represents the normalized signal power. The brain maps and curves for the gamma band are taken from Luo et al. (2007) with minor adaptations.

In the gamma band, the right amygdala showed a significant ERS for fearful but not for angry or neutral expressions. In the fear condition, significant gamma ERS peaked at 230–240 ms. In the theta band, the right amygdala also showed a significant ERS for fearful but not for angry or neutral expressions. In the fear condition, significant theta ERS peaked at around 230–240 ms. A *t*-test on the ERS peak latency revealed no significant difference between the two bands [*t*(1,14) = 1.7, *p* > 0.111]. See **Figure 1** for amygdala ERS in the two bands.

### THE VISUAL CORTEX

In the gamma band, the visual cortex (BA 17/18/19/37) showed significant ERS and had a similar spatiotemporal profile for the angry, fearful and neutral expressions. In the fear, angry and neutral conditions, significant ERS all peaked at 140–150 ms. In the theta band, the visual cortex (BA 17/18/19/37) also showed significant ERS and had a similar spatiotemporal profile for the angry, fearful, and neutral expressions. In the fear condition, significant ERS peaked at 200–210 ms. In the anger condition, significant ERS peaked at around 260–270 ms after stimulus onset. In the neural condition, significant ERS peaked at around 250–260 ms.

**FIGURE 2:**
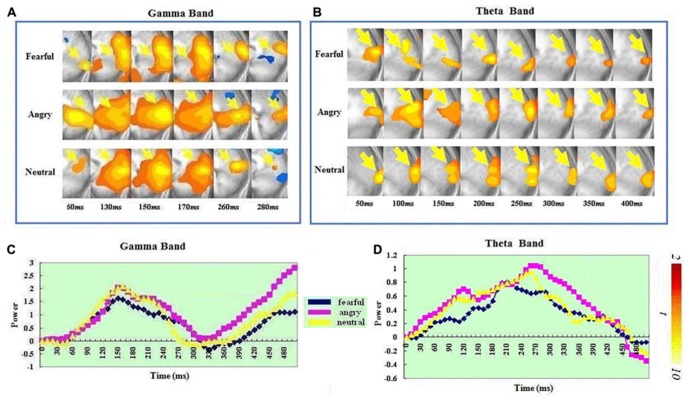
**Spatiotemporal profiles for the visual cortex in the gamma and theta bands.** The brain map series **(A,B)** indicate the ERS development in specific brain areas with the left hemisphere is on the right and the right hemisphere is on the left. The curves **(C,D)** represent temporal profiles obtained from a peak voxel in a specified area in the three conditions respectively. The time courses are obtained from the normalized power value in each window. The blue curve refers to responses to fearful, the purple to angry and the yellow to neutral expressions. The X-axis represents the development of time (ms) with an increment of 10 ms for each time segment. The Y-axis represents the normalized signal power. The brain maps and curves for the gamma band are taken from Luo et al. (2007) with minor adaptations.

A *t*-test on the ERS peak latencies revealed a significant difference between the two bands for all the three conditions: fear: *t*(1,14) = 7.332, *p* < 0.0001); anger: *t*(1,14) = 3.969, *p* < 0.001 and neutral: *t*(1,14) = 4.139, *p* < 0.001). In all the three conditions, the ERS peak was earlier in the gamma than in the theta band. See **Figure 2** for visual ERS in the two bands.

### IFG-INSULA

**FIGURE 3:**
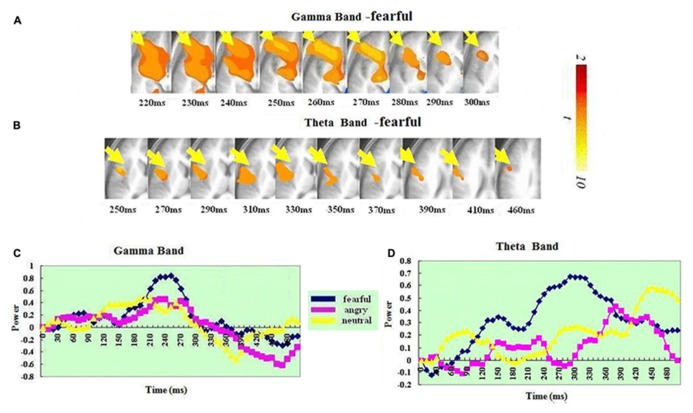
**Spatiotemporal profiles for IFG-insula in the in the gamma and theta bands.** The brain map series **(A,B)** indicate the ERS development in specific brain areas with the left hemisphere is on the right and the right hemisphere is on the left. The curves **(C,D)** represent temporal profiles obtained from a peak voxel in a specified area in the three conditions respectively. The time courses are obtained from the normalized power value in each window. The blue curve refers to responses to fearful, the purple to angry and the yellow to neutral expressions. The X-axis represents the development of time (ms) with an increment of 10 ms for each time segment. The Y-axis represents the normalized signal power. The brain maps and curves for the gamma band are taken from Luo et al. (2007) with minor adaptations.

In the gamma band, a significant ERS in the right IFG-insula (BA 47, 13) was seen in the fear condition but not in the anger or neutral condition. It peaked at 240–250 ms. In the theta band, a significant ERS in the right IFG-insula (BA 44, 47, 13) was also seen in the fear condition but not in the anger or neutral condition. It peaked at 330–340 ms. A *t*-test on the ERS peak latencies showed that the ERS peak was earlier in the gamma than in the theta band [t(1,14) = 3.013, *p* < 0.009]. See **Figure 3** for IFG/insula ERS in the two bands.

### OFC–ACC

In the gamma band, ERS in the OFC (BA 10) was found for just the anger condition. It peaked at 200–210 ms. In the theta band, a significant ERS in the left OFC–ACC (BA 10 and 32; mostly OFC-BA10) was also seen in just the anger condition but not in the fear or neutral condition. It peaked at 440–450 ms. A *t*-test on the ERS peak latency showed that ERS peak was earlier in the gamma than in the theta band [t(1,14) = 7.826, *p* < 0.0001].

## DISCUSSION

The present study examined theta band activity in response to emotional expressions and its relationship with gamma band activity using MEG and an advanced beamformer source localizing technique. We found that areas including the amygdala, visual cortex, and frontal cortex showed significant ERS in both the gamma and the theta band. In addition, with the amygdala, activity in both bands shared a similar temporal profile. However, within visual and two regions of frontal cortex, ERS peaked significantly earlier in the gamma relative to the theta band.

The current findings challenge the traditional view that high and low frequencies are spatially distinctly localized to neocortex and evolutionarily more ancient structures respectively (MacLean, 1990; Michel et al., 1992; Robinson, 2000, 2001; Knyazev and Slobodskaya, 2003; Knyazev et al., 2004). The current data show a notable overlap in spatial location within both amygdala and regions of cortex. However, while the peak in activity within both the theta and gamma bands was similar within the amygdala, the theta peak was significantly slower within regions of cortex. These findings will be discussed at a region specific level below.

### AMYGDALA

There have been previous reports showing that emotional processing is associated with both gamma (Müller et al., 1999; Taylor et al., 2000; Keil et al., 2001; Oya et al., 2002; Luo et al., 2007) and theta band oscillations (Paré and Collins, 2000; Aftanas et al., 2001; Paré et al., 2002; Seidenbecher et al., 2003). Moreover, neuronal oscillations in the amygdala have been observed in the gamma band in humans (Oya et al., 2002; Luo et al., 2007) and in the theta band in animals (Seidenbecher et al., 2003; see Paré et al., 2002 for a review). Our study is the first showing both gamma and theta synchronization in the amygdala in response to fearful faces within the same study. Notably, the spatial location of the amygdala ERS in the two bands was homologous (See **Figure 1**). Moreover, the ERS peak time was statistically similar for the two bands: 230–240 ms after stimulus onset. Such high spatial-temporal consistency in the amygdala suggests a functional association in neuronal oscillations in the two bands, and even potentially their interaction (though this remains to be empirically demonstrated) within the amygdala. As such our data extend previous findings of a relationship between activity in the gamma and theta bands in the hippocampus (e.g., Bragin et al., 1995; Lisman and Idiart, 1995; Jensen and Lisman, 1996; Chrobak and Buzsáki, 1998; Jensen and Tesche, 2002; Rizzuto et al., 2003).

It is interesting to note that the amygdala was the only region where the ERS peak was the same for the two bands. The co-presence of theta and gamma band activities in the brain has been suggested to reflect the interdependency of the two different oscillation frequencies and the need to integrate multiple layers of information for multiple time-scale control of neuronal spikes within and across structures (Belluscio et al., 2012). The co-presence of the oscillations in the two bands with a similar time course possibly reflects the integration of multiple sub-processes within the amygdala’s response to an emotional stimulus.

### VISUAL CORTEX

Significant ERS in visual cortex was found for both the theta and the gamma band and was spatially homologous for all three conditions (see **Figure 2**). Gamma band oscillations have been consistently reported in visual cortex (Fries et al., 1997; Logothetis et al., 2001; Bichot et al., 2005; Womelsdorf et al., 2006; Luo et al., 2007). Little has been reported on theta synchronization in visual cortex though there has been at least one study reporting both theta and gamma band activity in visual cortex (Lee et al., 2005).

Temporally, the ERS peak came significantly later in the theta (200–270 ms) than in the gamma band (140–150 ms). This suggests a functional difference between neuronal activities within the visual cortex over this time course. Neuronal synchronization in visual cortex is thought to be associated with visual feature binding (Crick and Koch, 1990; Fries et al., 1997; Reynolds and Desimone, 1999). It has also been shown that visual cortex is important for visual working memory (Supèr et al., 2001; see Supèr, 2003 for a review). Interestingly, theta band oscillation in visual cortex is involved in working memory (Lee et al., 2005). It can therefore be postulated that the peak latency difference in the visual ERS in the two bands reflects a serial process of feature binding for forming a coherent representation and then active maintenance or storage of the visual representation in visual working memory.

### THE FRONTAL CORTEX

**FIGURE 4:**
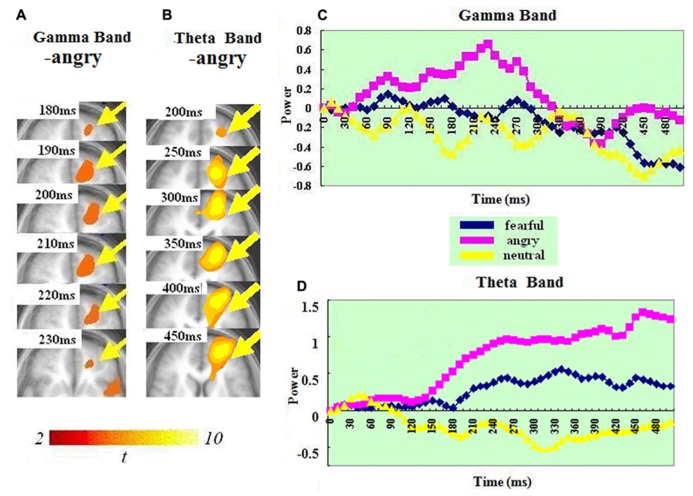
**Spatiotemporal profiles for OFC–ACC in the gamma and theta bands.** The brain map series **(A,B)** indicate the ERS development in specific brain areas with the left hemisphere is on the right and the right hemisphere is on the left. The curves **(C,D)** represent temporal profiles obtained from a peak voxel in a specified area in the three conditions respectively. The time courses are obtained from the normalized power value in each window. The blue curve refers to responses to fearful, the purple to angry and the yellow to neutral expressions. The X-axis represents the development of time (ms) with an increment of 10 ms for each time segment. The Y-axis represents the normalized signal power. The brain maps and curves for the gamma band are taken from Luo et al. (2007) with minor adaptations.

Our results revealed significant ERS in two regions of frontal cortex (left OFC–ACC and right IFG-insula). This was present in both bands (see **Figures 3** and **4**). In both regions, the ERS peaked earlier in the gamma (OFC–ACC: 200–210ms; IFG/insula: 240–250ms) than in the theta band (OFC–ACC: 440–450ms; IFG/insula: 330–340 ms). Frontal cortex is deemed important in cognitive and emotional integration, response regulation and decision-making (Ochsner and Gross, 2005). It is suggested that gamma band synchrony is important for fast and transient integration of distributed neuronal processes into the formation of a coherent representation of the stimulus (Singer, 1999; Varela et al., 2001). Therefore it is possible that the frontal gamma ERS observed in the present study reflects cognitive and emotional integration, which is perhaps a prelude for decision-making. The frontal theta ERS, which came after the integration indexed via the gamma band may reflect response regulation and decision-making. In line with this, there are previous reports of a relationship between frontal theta activity and action regulation (Luu and Tucker, 2001; Luu et al., 2004; Makeig et al., 2004; Kirmizi-Alsan et al., 2006). The fact that the theta ERS power in OFC–ACC peaked at 440–450 ms – just the time approaching the response window (see **Figure 4**) possibly also suggests an involvement in response preparation and decision-making.

To sum up, our data indicate that both gamma and theta bands index activity occurring in overlapping regions of amygdala as well as frontal and visual cortex in response to emotional stimuli. Within the amygdala, activity in both bands shared a similar temporal profile perhaps reflecting interaction between the activities eliciting activity in both bands. In contrast, within frontal and visual cortical regions there was a clear difference in temporal profile of activity in the two bands. This may reflect either different, but related, functional processes or, perhaps more probably, different computational components of the same functional process within these regions.

## Conflict of Interest Statement

The authors declare that the research was conducted in the absence of any commercial or financial relationships that could be construed as a potential conflict of interest.

## Footnotes

1 http://afni.nimh.nih.gov

2 http://biowulf.nih.gov/
